# Effects of Exercise on Sarcopenia and Frailty in Haemodialysis Patients: A Systematic Review

**DOI:** 10.3390/medicina61122204

**Published:** 2025-12-12

**Authors:** Elisa María Garrido-Ardila, Miguel Ángel Castro Lemus, María del Valle Ramírez-Durán, María Jiménez-Palomares, María Victoria Martín Hidalgo-Barquero, Blanca González-Sánchez, Juan Rodríguez-Mansilla

**Affiliations:** 1 ADOLOR Research Group, Department of Medical-Surgical Therapy, Medicine Faculty and Health Sciences, University of Extremadura, 06006 Badajoz, Spain; egarridoa@unex.es (E.M.G.-A.); jrodman@unex.es (J.R.-M.); 2 Physiotherapist, Medicine Faculty and Health Sciences, University of Extremadura, 06006 Badajoz, Spain; micastrol@alumnos.unex.es; 3 Department of Nursing, Plasencia University Centre, University of Extremadura, 10600 Plasencia, Spain; valleramirez@unex.es; 4 Nephrology Department, Badajoz University Hospital, 06006 Badajoz, Spain; mariavictoria.martin@salud-juntaex.es

**Keywords:** haemodialysis, chronic kidney disease, physiotherapy, exercise, physical performance, sarcopenia, frailty, systematic review

## Abstract

*Background and Objectives*: Chronic kidney disease is characterized by the progressive loss of functioning nephrons due to structural and functional alterations in the kidneys. It is clinically defined through the presence of a glomerular filtration rate below 60 mL/min/1.73 m^2^ or persistent kidney damage lasting at least three months. Patients undergoing haemodialysis frequently present with sarcopenia and frailty. The aim of this study was to evaluate the effects of intradialytic exercise on sarcopenia and frailty in individuals with chronic kidney disease. *Materials and Methods*: A systematic review was conducted in accordance with PRISMA guidelines. Electronic searches were executed in PubMed, PEDro, Scopus, and Dialnet. Eligible studies included adults (≥18 years) on haemodialysis who engaged in exercise interventions compared with passive control groups. Exclusion criteria included any conditions conflicting with the inclusion criteria, systematic reviews, study protocols, and articles not meeting the PICO framework or contradictory to the inclusion criteria. Outcomes of interest were sarcopenia and frailty, assessed through measures of physical function and muscle strength. Methodological quality was appraised using the PEDro scale, and risk of bias was evaluated with the Cochrane Risk of Bias tool. *Results:* Fifteen studies met the inclusion criteria. Most interventions consisted of aerobic training, resistance training, or combined exercise programs. Across studies, exercise interventions consistently improved physical function and muscle strength, although no significant effects on body composition were observed. *Conclusions*: This systematic review provides evidence that intradialytic exercise may produce clinically relevant improvements in sarcopenia by enhancing muscle strength and functional performance, as measured by tests such as the sitting-to-standing test. These results suggest that intradialytic exercise could be beneficial for patients with chronic kidney disease.

## 1. Introduction

Chronic kidney disease (CKD) is characterized by the progressive loss of functioning nephrons resulting from structural and functional alterations of the kidneys or persistent renal damage lasting three months or longer [1]. According to the glomerular filtration rate, kidney disease is commonly classified into two main stages: renal insufficiency and advanced kidney disease [2].

Its prevalence increases with age, as shown by the EPIRCE study (Epidemiología de la Insuficiencia Renal Crónica en España—Epidemiology of Chronic Kidney Disease in Spain), which reported a rate of 1.6% in individuals over 64 years, further amplified by comorbid chronic conditions [2]. Diabetes mellitus is the primary etiological factor in the development of CKD, although there are additional determinants including susceptibility factors (age, family history, ethnicity, and low educational level), indicator factors (urinary tract obstruction, nephrolithiasis, and recurrent urinary tract infections), progression factors (proteinuria, anaemia, uncontrolled diabetes, among others), and end-stage determinants (delayed referral to nephrology and hypoalbuminemia) [1,3].

CKD progression is often asymptomatic in early stages but eventually leads to initial symptoms including nocturia and polyuria, and progressive stages are associated with nausea, vomiting, anorexia, fatigue, weakness, sleep disturbances, oliguria, impaired cognition, muscle cramps, peripheral oedema, pruritus, chest pain secondary to uremic pericarditis, and dyspnoea caused by fluid overload and hypertension [1,4].

Within this context, the vulnerability of present patients with chronic kidney disease has emerged as a critical concern with two conceptualizations. One regards sarcopenia which is condition frequently associated with chronic kidney disease. The most up-to-date conceptualisation, established by the European Working Group on Older People (EWGSOP), defines sarcopenia as geriatric syndrome which symptoms include low muscle mass, reduced muscle strength and low performance [5]. In addition, The International Classification of Diseases (ICD-10) code for sarcopenia recognizes it as a progressive and generalized muscle disorder characterized by the loss of muscle mass, strength, and function [6]. It is characterised by phenotypic manifestations such as slow gait speed, unintentional weight loss, low physical activity, fatigue, and muscle weakness. The other describes frailty as the cumulative effect of cellular deficits that progressively compromise organ systems, positioning frailty as a biomarker of biological aging [7]. From the perspective of the joint European action on prevention and management of frailty (ADVANTAGE), frailty is understood as a clinical state of vulnerability that restricts independence and is potentially fatal in the face of stressors [8].

In CKD, frailty is strongly associated with functional dependency and comorbidity, both of which markedly worsen prognosis, as dependency arises from impairments in mobility and in the ability to perform instrumental and basic activities of daily living that require adequate physical and cognitive function, thereby linking frailty closely to declines in both physical performance and cognitive status [8,9]. Sarcopenia, defined as the age-related loss of skeletal muscle mass, is intrinsically linked to frailty and dependency, and in CKD it further exacerbates functional impairment, reduces physical performance, and increases disability [5,9].

A growing body of evidence supports physical exercise as a therapeutic strategy in patients with CKD, given its association with reduced mortality and improved graft or transplant survival [5]. Exercise interventions consistently demonstrate significant benefits in functional capacity and aerobic performance [5], and current research emphasizes the importance of initiating such programs in the early stages of CKD, as declines in physical function closely parallel reductions in glomerular filtration rate, while sedentary behavior has been identified as a potential contributor to disease progression [10]. Among available approaches, supervised intradialytic aerobic exercise is most frequently recommended [11,12], although resistance training [11] and complementary modalities such as yoga or qi gong (which consists of a mindfulness technique, steady deep breathing, slow body movement, and relaxing one’s posture) [13] have also shown promise [12]. The mentioned scientific literature shows that there is an increasingly implementation of physical exercise programmes in haemodialysis patients and this systematic review is justified by the need to assess their effectiveness based on the analysis of the most recent studies. In light of these considerations, the objective of this study was to analyze the effects of intradialytic physical exercise on sarcopenia and frailty in patients with CKD.

## 2. Materials and Methods

### 2.1. Study Design

This systematic review was carried out following the PRISMA statement [14] and was registered in PROSPERO with the registration number CRD420251125939 the protocol is available at https://www.crd.york.ac.uk/PROSPERO/view/CRD420251125939 (accessed on 15 June 2025).

### 2.2. Search Strategy

Relevant studies were identified through systematic searches in Scopus, PubMed, Dialnet, and PEDro (Physiotherapy Evidence Database). To ensure precision and specificity, descriptors were extracted from the DeCS (Health Sciences Descriptors) and MeSH (Medical Subject Headings) thesauri. Keywords were combined using the Boolean operators AND and OR. The search in the databases was performed during the month of May 2025.

The MeSH terms employed included: hemodialysis, chronic kidney disease, hand grip strength, short physical performance battery, muscle strength, physiotherapy, sarcopenia, exercise, body mass index, functional physical performance, end-stage renal disease, frailty, Fried phenotype, weakness, and gait speed test. These terms were combined using the Boolean operators AND and OR in Scopus, PEDro and PubMed, with queries adapted to the MeSH library syntax.

For the Dialnet database, a simplified strategy was applied by combining the terms hemodialysis and exercise. Scopus and Pubmed allowed to enter filters and they were: publication date—10 years; article type—clinical trial, randomized clinical trials; and article language—English, Spanish. Table 1 reflect the search strategies entered into these databases.

### 2.3. Eligibility Criteria

The selection criteria were established following the PICO’s framework. The inclusion criteria were:

- Population: Participants with chronic kidney disease, over 18 years old and receiving haemodialysis treatment.
- Intervention: Interventions that carried out intradialytic exercise programmes.
- Comparison: Control groups where the participants continued with their haemodialysis treatment but did not performed exercise during haemodialysis.
- Outcome measures: Variables measuring sarcopenia and frailty. Frailty was assessed using variables related to physical function, including the 6 min walk test (6 MWT), the Short Physical Performance Battery (SPPB), and gait speed. Sarcopenia was evaluated through body composition and strength-related measures, such as body mass index (BMI), handgrip strength, and lower limb strength. The sit-to-stand test was considered the most specific functional measure for sarcopenia, while also serving as an indicator of frailty.
- Type of studies: clinical trials and randomized controlled trials published in the last 10 years in Spanish or English, which included at least two study outcome measures.

Exclusion criteria included interventions with patients on peritoneal dialysis, participants under 18 years old, studies which design was systematic reviews or study protocols, articles not meeting the PICO framework and articles published in other languages than English or Spanish.

### 2.4. Selection of Studies

The study selection process was carried out in several stages. First, keywords were entered into the selected databases. Titles were screened to identify potentially relevant articles, which were then exported to the Zotero reference manager. Duplicates were removed, and abstracts of the remaining records were reviewed. Articles that met the inclusion criteria were retrieved for full-text assessment. Final inclusion was determined based on a complete reading of the articles according to the predefined eligibility criteria. The selected studies were subsequently evaluated for methodological quality and risk of bias.

The following data were obtained from the studies included in the review: author, mean age, sample size, type of intervention, assessment tools and results. These data were compiled in a standard table. We also collected the data and assessed the methodological quality and the risk of bias of the studies.

All potential full-text articles were independently retrieved and evaluated by two reviewers. The level of agreement between the two reviewers was not specifically calculated. However, any disagreement on inclusion or exclusion of the full-text articles was discussed and resolved.

### 2.5. Methodological Quality Analysis

The methodological quality of the included studies was assessed using the PEDro (Physiotherapy Evidence Database) scale [15]. This instrument consists of 11 items rated as “yes” (Y) or “no” (N). As the first item refers to external validity and is not included in the overall score, the maximum possible score is 10. Items 2–9 evaluate internal validity, while items 10 and 11 assess whether the statistical information is sufficient to allow interpretation of the results [15]. According to the PEDro classification, scores above 5 indicate high methodological quality (6–8: good; 9–10: excellent), scores of 4–5 indicate moderate quality (regular), and scores below 4 indicate low quality (poor).

### 2.6. Risk of Bias Analysis

Risk of bias was evaluated using the Cochrane Risk of Bias Tool, adapted by Higgins and Altman [16]. This instrument assesses potential bias across six domains of clinical trial methodology: random sequence generation, allocation concealment, blinding of participants and personnel, blinding of outcome assessors, incomplete outcome data, selective outcome reporting, and other potential sources of bias. Each domain is rated to reflect the degree of bias risk, thereby providing a structured evaluation of the overall reliability of study findings.

## 3. Results

**Figure 1:**
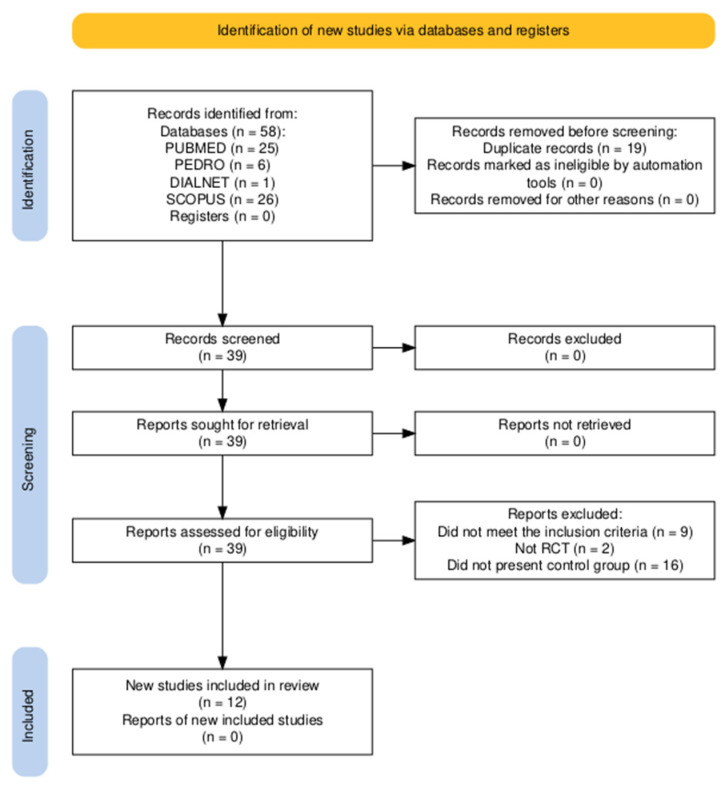
PRISMA flow diagram.

The literature search was conducted between November 2024 and August 2025. A total of 58 studies were obtained from the search of all databases. Duplicate records were excluded, and 12 studies were finally selected. The study selection process is shown in the PRISMA flow chart (Figure 1).

The characteristics of these studies are collected and detailed in the following table (Table 2: Studies collected).

### 3.1. Sociodemographic and Methodological Characteristics

The studies included in this systematic review encompassed a total of 684 participants with CKD undergoing hemodialysis. Sample sizes ranged from 12 participants in the study by Assawasaksakul et al. [25] to 123 participants in the study by Suhardjono et al. [19]. All studies were randomized clinical trials, except for Yabe et al. [20], which employed a non-randomized design. Among the randomized trials, 10 studies [17,18,19,21,22,23,24,25,26,27,28] explicitly described the randomization procedure, and 8 [17,18,19,22,23,24,25,28] reported adequate concealment of participant allocation.

Participants were adults with CKD who had been receiving hemodialysis for 3–6 months, typically three sessions per week, each lasting approximately four hours. The mean age of participants across studies was 56 years, with a predominance of male patients (373 men, 54%) compared with female patients (311 women, 45%).

The variables assessed across the studies primarily reflected functional capacity, daily physical activity, body composition, and muscular strength, and, in one case, muscle architecture. This focus aligns with the characteristic physical and muscular impairments observed in hemodialysis patients with sarcopenia and frailty. Physical function was evaluated through the 6 min walk test, sit-to-stand test, Short Physical Performance Battery, and gait speed test. Muscle strength was assessed using handgrip strength and static lower-limb strength, whereas body composition was measured using body mass index. In one study, muscle architecture was additionally analyzed via ultrasound imaging (Krase et al. [18]).

With respect to exercise protocols the review identified four aerobic exercise programs (Krase et al. [18]; Vogiatzaki et al. [24]; Cardoso et al. [17]; Abdo et al. [27]), one resistance training program (Zhang et al. [22]), and seven combined aerobic and resistance protocols (Assawasaksakul et al. [25]; Jamshidpour et al. [23]; Suhardjono et al. [19]; Yabe et al. [20,21]; Yeh et al. [28]; Michou et al. [26]). Intervention duration ranged from 2 to 12 months, except for the study by Krase et al. [18], which lasted one month. All programs were performed during the first two hours of hemodialysis and typically scheduled on alternate days. Exercise intensity was prescribed according to maximum heart rate (HRmax) and the Borg Rating of Perceived Exertion (RPE) scale, with continuous monitoring of hemodynamic parameters and potential adverse events to ensure patient safety.

The aerobic protocols, implemented in four studies [17,18,24,27], were supervised by exercise specialists, nurses, or physiotherapists and lasted between 20 and 60 min. All involved intradialytic cycling using a cycle ergometer at 60–70% HRmax and a Borg RPE of 10–14. Intensity was progressively increased throughout sessions or across the program duration. For instance, Krase et al. [18] prescribed cycling at 45 rpm in the first phase and 60 rpm in the second, whereas Cardoso et al. [17] progressed from 60–63% HRmax (Borg 10–11) during the first six weeks to 64–75% HRmax (Borg 12–13), extending session duration by five minutes weekly until reaching 45 min. Abdo et al. [27] incorporated a warm-up phase with joint mobility exercises, a main phase of cycling at 50–70% HRmax, and a cool-down period.

A single resistance training program was reported by Zhang et al. [22]. The intervention, administered by nephrology staff, lasted 40–50 min and comprised four exercises—biceps curl, shoulder flexion, straight leg raise, and leg extension—performed with wrist and ankle weights. The program included three phases: warm-up, main training, and cool-down. Warm-up and cool-down consisted of five minutes of stretching at Borg 8–10, while the main phase included three sets of 11–12 repetitions, performed twice weekly for the first four weeks (Borg 10–13) and three times weekly from weeks five to twelve.

Seven studies implemented combined aerobic and resistance programs [19,20,21,23,25,26,28]. These were conducted by cardiologists, sports physicians, physiotherapists, or nurses trained in physiotherapist-designed protocols, and in one study [26], specialized exercise trainers supervised the sessions. The aerobic component typically lasted 20–30 min, while resistance exercise duration was not explicitly defined. Most studies (n = 5) [21,23,25,26,28] incorporated a structured sequence of warm-up, aerobic cycling, and strength training, whereas two [20,23] omitted the warm-up. Program components generally consisted of a 5 min warm-up, 20–60 min of cycling at Borg 11–14, and three sets of 8–15 repetitions at Borg 11–13 targeting lower-limb movements (leg extension, abduction, flexion, and straight leg raises). Michou et al. [26] specified 40–60 s of recovery between sets.

Individualized progression strategies were also reported. Assawasaksakul et al. [25] prescribed progressive cycling from 40 rpm for 10 min (0–4 W) to 80–100 rpm at 10 W, followed by a resistance phase exceeding 10 W (Borg 13) and a cool-down phase matching the warm-up parameters. Yeh et al. [28] progressively increased ergometer resistance every 10 min, maintaining intensity at Borg 12–14. Jamshidpour et al. [23] applied a progressive workload from Borg 11 to 15, extended session duration by five minutes up to 45 min and performed strength training at 60% of one-repetition maximum (1RM). Suhardjono et al. [19] also incorporated progressive cycling, increasing intensity from 40–60% HRmax in the initial phase to 60–80% HRmax in later months.

The majority of studies used SPSS software for data analysis. All included comparisons between experimental and control groups and reported effect sizes and measures of variability for the outcomes examined [17,18,19,20,21,22,23,24,25,26,27,28].

### 3.2. Results Related to Variables and Study Outcomes

Overall, the reviewed studies demonstrated significant improvements in physical function, handgrip strength, and lower-limb strength among participants in the exercise intervention groups, whereas control groups generally showed no change or a decline in these variables.

#### 3.2.1. Physical Function

The 6 min walk test: Significant improvements were observed in seven studies. The aerobic protocols of Cardoso et al. [17] (412.74 m → 483.74 m; *p* = 0.007), Vogiatzaki et al. [24] (442 m → 481 m; *p* = 0.02), and Krase et al. [18] (404.3 m → 468.1 m; *p* = 0.001) demonstrated substantial gains in walking distance. Combined aerobic–resistance interventions also improved performance, including Jamshidpour et al. [23] (249.53 m → 338.97 m; *p* < 0.05), Michou et al. [26] (427 m → 468 m; *p* < 0.05), and Yeh et al. [28] (345.98 m → 395.77 m; *p* < 0.001). The resistance program by Zhang et al. [22] produced a smaller but significant improvement (406 m → 409.49 m; *p* = 0.026). On average, aerobic interventions increased 6MWT distance by 57.9 m, combined protocols by ≈60 m, and the resistance protocol by ≈3 m, indicating that aerobic and combined programs elicited the largest functional gains.

Sit-to-stand test: Significant improvements were reported in three studies. The resistance program by Zhang et al. [22] reduced STS-10 time (25.20 s → 23.8 s; *p* < 0.001). The aerobic program by Krase et al. [18] increased repetitions in STS-60 (23.45 → 25.85; *p* = 0.001). Among combined protocols, Assawasaksakul et al. [25] reported an STS-10 improvement (11.10 s → 10.33 s; *p* < 0.05), whereas Michou et al. [26] found a decline (22.40 s → 24.80 s; *p* < 0.005). Yeh et al. [28] recorded gains in both STS-10 (30.30 s → 24.67 s; *p* < 0.01) and STS-60 (19.59 → 20.04 repetitions; *p* < 0.001). Collectively, aerobic, resistance, and combined programs improved lower-body functional performance.

Short Physical Performance Battery (SPPB): The combined aerobic–resistance program by Yabe et al. [21] significantly increased mean SPPB scores (10 → 12; *p* < 0.005), whereas Yabe et al. [20] found no statistically significant change.

Gait Speed: Mixed results were reported. Yabe et al. [20] observed a decline in gait speed in the control group (1.33 m/s → 1.19 m/s; *p* < 0.005), while Suhardjono et al. [19] reported no significant differences following intervention.

#### 3.2.2. Muscle Strength

Handgrip Strength: Four studies reported significant results. The resistance program by Zhang et al. [22] increased grip strength (25.71 kg → 26.53 kg; *p* = 0.03), and the aerobic program by Krase et al. [18] improved from 29.14 kg to 30.64 kg (*p* = 0.02). In combined programs, Michou et al. [26] observed an increase (26.59 kg → 28.61 kg; *p* < 0.005), whereas Yabe et al. [20] noted a decrease in the control group (26.72 kg → 23.9 kg; *p* < 0.005). Overall, the resistance and aerobic interventions increased grip strength by 0.8–1.5 kg, while combined programs yielded a net gain of approximately 2 kg in the intervention groups.

Lower-Limb Strength: Among the aerobic protocols, Cardoso et al. [17] reported a non-significant improvement (59.72 kg → 66.6 kg; *p* = 0.15), whereas Abdo et al. [27] found a significant increase in quadriceps strength (22.63 kg → 24.26 kg; *p* = 0.001). In the combined protocols, Jamshidpour et al. [23] and Suhardjono et al. [19] demonstrated significant gains, while others (Yabe et al. [20,21]) reported no statistical differences. Collectively, aerobic and combined interventions improved lower-limb strength, with the most consistent effects observed in quadriceps performance.

Body Composition and Muscle Architecture

Three studies evaluated body mass index (BMI)—Suhardjono et al. [19], Assawasaksakul et al. [25], and Vogiatzaki et al. [24]—but none found significant changes. The ultrasound analysis of muscle architecture by Krase et al. [18] revealed a significant reduction in vastus lateralis thickness (*p* = 0.001), indicating loss of muscle mass despite functional improvement.

#### 3.2.3. Frailty

Only one study explicitly assessed frailty, using the Older Adult Health Check Questionnaire (QMCOO). The combined aerobic–resistance program by Yabe et al. [20] did not produce significant improvements among frail participants who required walking assistance.

### 3.3. Methodological Quality of the Articles

The findings related to the assessment of methodological quality, according to the PEDro scale, are presented in Table 3. It should be noted that a negative response does not automatically imply that the study lacks the characteristic in question. Rather, it indicates that, despite a detailed review of the article, the specific requirement could not be clearly identified in the text.

### 3.4. Risk of Bias

The risk of bias was assessed using the Cochrane Risk of Bias tool. The results are presented in the following table (Table 4).

The main risk of bias identified in the reviewed studies is related to the blinding of participants and personnel, which is difficult to achieve in exercise intervention studies. This domain presents a high risk of bias in all studies. The domain of random sequence generation presents a high risk of bias in one study [20], and the domain of allocation concealment presents a high risk of bias in three studies [20,26,27]. The rest of the domains show a low risk of bias in all studies

## 4. Discussion

The aim of this review was to analyze the effects of physical exercise on sarcopenia and frailty in patients undergoing hemodialysis. The exercise programs reviewed encompassed three main modalities: resistance training, aerobic exercise, and combined training incorporating both components. The variables that reflected the impact of these programs included measures of physical fitness (6MWT, STS, SPPB, and gait speed), muscle strength (handgrip and lower-limb strength, both assessed with a dynamometer), and body composition. Physical fitness variables are more closely related to the assessment of frailty, whereas muscle strength and body composition are more directly associated with sarcopenia. The STS test, in particular, serves as an indicator of both conditions.

The results of this review suggest that exercise, across its various modalities, exerts a positive effect on physical function. This benefit is most evident in the distance covered during the 6MWT and the ability to rise from a chair, with additional improvements detected in gait speed and overall physical performance. In terms of strength outcomes, the most reliable measurements were obtained from protocols including handgrip strength, which generally improved across studies. Regarding body composition, this review cannot draw definitive conclusions, as the available evidence was inconsistent and inconclusive. It is also noteworthy that the absence of exercise had a detrimental impact on both physical fitness and muscle strength. The improvements reported across the exercise protocols included in this review are consistent with previous findings in the literature, including the systematic reviews by Zhang et al. [29] and Bündchen et al. [30], and the clinical trials conducted by Kim et al. [31], Torres et al. [32], and Chaoverin et al. [33].

The systematic review by Bündchen et al. [30] on the effects of exercise in end-stage renal disease demonstrated that intradialytic exercise improves functional capacity, which is closely linked to frailty, and enhances isometric quadriceps strength, an indicator of sarcopenia, findings that are consistent with the present review. Bündchen et al. [30] also identified the 6MWT as the most reliable measure of functional capacity, one of the most frequently used and strongly supported tests in this review. Furthermore, they reported that combined exercise improved both psychosocial well-being and physical outcomes. Similarly, the review by Zhang et al. [29], which examined the effects of intradialytic exercise on physical performance, nutrient intake, and quality of life, found that resistance exercise produced significant improvements in the STS-30, 6MWT, and handgrip strength tests—all of which were also included in the resistance-based protocols analyzed in the present review.

With respect to physical function, other studies in the literature [31,32] also reported improvements in the 6MWT, consistent with the results of the studies included here. Kim et al. [31] developed an aerobic exercise program similar in design to those reported by Krase et al. [18], Cardoso et al. [17], and Vogiatzaki et al. [24]. The program by Kim et al. [31] lasted three months, aligning with the duration range of the interventions analyzed in this review, and the session structure—comprising warm-up, main exercise, and cool-down phases—also matched the framework of comparable aerobic programs [17,18,24]. However, Krase et al. [18] and Vogiatzaki et al. [24] did not follow this phased structure, beginning the sessions directly with the main activity and progressively increasing intensity. The Vogiatzaki et al. [24] protocol was more similar to Kim et al. [31], as it included a warm-up phase but no cool-down. The warm-up phase in both Kim et al. [31] and Vogiatzaki et al. [24] involved joint mobility and stretching exercises, whereas Krase et al. [18] began directly with progressive cycling without a preparatory warm-up. Interestingly, Krase et al. [18] achieved a greater increase in the distance walked in six minutes compared with Vogiatzaki et al. [24] and reported improvements in handgrip strength. These findings suggest that progressive intensity training may yield greater benefits than programs emphasizing pre-exercise mobility and stretching.

In terms of exercise intensity, Kim et al. [31] employed very low intensities (Borg 7–8) compared with the higher intensities applied in the aerobic programs reviewed here (Borg 10–13). It is plausible that had Kim et al. [31] implemented a progressive increase in intensity up to the target level of the main activity, greater aerobic benefits would have been achieved.

The Kim et al. [31] program was designed by nephrologists and nurses, whereas the protocols included in this review [17,18,24] involved physiotherapists, nurses, and exercise specialists. Improvements in physical function and muscular strength were greater in protocols led by physiotherapists and exercise professionals, suggesting that professional expertise may influence the magnitude of outcomes. Thus, interventions supervised by physiotherapists and exercise professionals may yield superior improvements in both frailty and sarcopenia [17,18].

In terms of results, Kim et al. [31] observed improvements in gait speed and SPPB scores. Although the aerobic programs [17,18,24] did not assess these variables, the combined exercise program by Yabe et al. [20] included both measures and reported improvements in SPPB scores, while noting that the absence of exercise contributed to a decline in gait speed. These findings align with the conclusions of Bündchen et al. [29], who associated functional capacity (measured by peak oxygen uptake, quality of life, and cardiopulmonary test duration) with combined exercise and confirmed its positive impact on physical outcomes.

The analysis of strength training programs was supported by comparisons with other trials in the literature, including Chaoverin et al. [33] and Torres et al. [32], alongside the resistance protocol of Zhang et al. [22] and the combined exercise programs analyzed in this review. In terms of duration and frequency, the studies by Chaoverin et al. [33] and Torres et al. [32] were comparable to Zhang et al. [22] and the other exercise modalities [17,18,19,20,21,23,24,25,26,27,28]. A notable distinction lies in how workload was quantified: Chaoverin et al. [32] employed one-repetition maximum (1RM) testing, Zhang et al. [22] used the Borg scale, and the combined protocols also relied on the Borg scale [19,20,21,23,25,26,28]. Only Jamshidpour et al. [23] employed a related measure—three-repetition maximum (3RM)—representing a lower load threshold than 1RM [23,33]. Jamshidpour et al. [23] reported improvements in leg muscle mass and STS performance, suggesting that both dosing strategies—1RM and 3RM—had positive effects on muscle mass, though 3RM may have greater impact on frailty through improved 6MWT performance, whereas 1RM may be more beneficial for both frailty and sarcopenia by enhancing STS outcomes.

The Chaoverin et al. [32] study also implemented progressive load increments, similar to the resistance and endurance programs in the present review [17,18,24], and achieved improvements in muscular function but not in body composition, consistent with our findings. Although Chaoverin et al. [32] used higher and progressively increased intensities, the overall pattern of improvement was similar to that observed in the reviewed studies. Conversely, two combined programs [25,28] did not employ resistance tools such as dumbbells or elastic bands; instead, they increased ergometer resistance, thereby excluding upper-limb work. The muscular stimuli generated by cycling differ from those produced by free weights, and the inertial forces of pedalling may alter the effective resistance experienced by the participant, potentially affecting training intensity.

The study by Torres et al. [32] provides a useful comparison with the protocols reviewed here. Their program was developed by the rehabilitation department and implemented by trained rehabilitation staff. It included various motor tasks organized into four sets of 20 repetitions, with additional loads applied when patients completed the exercises with ease. Upper-limb exercises were also performed in four series of repetitions. With this approach, Torres et al. [32] achieved improvements in BMI. The key differences between this protocol and those in the present review lie in the number of repetitions and load management. The maximum number of repetitions observed in the reviewed studies was 15, and resistance was determined subjectively using the Borg scale, whereas Torres et al. [32] prescribed 20 repetitions and adjusted load progressively based on the participant’s ease of execution, resulting in a more intense training regimen. Consequently, Torres et al. [32] achieved greater improvements in body composition compared with the studies included in this review, likely due to the higher training intensity and workload, which may have produced more pronounced physiological adaptations.

The results of the methodological quality assessment and risk of bias analysis support the reliability of the improvements reported in the studies included in this systematic review, as 10 out of 12 articles demonstrated good methodological quality and a low risk of bias. Regarding the strengths of this review, it is worth highlighting its level of contemporaneity, as it includes studies published within the last ten years and characterized by methodological homogeneity in terms of study groups and measured variables. Furthermore, the inclusion of a passive control group in several trials allowed for a more accurate determination of the true effects of exercise on sarcopenia and frailty. Finally, by analyzing different types of exercise programs, this review provides insight into which modalities may be most beneficial for this patient population.

### Limitations of the Review

This review has several limitations. The heterogeneity of exercise protocols and outcome measures made difficult the analysis of the studies and precluded the performance of a meta-analysis. Regarding the databases used, although PEDro is widely recognized in the international literature, its scope is restricted to the field of physical therapy, which is the subject of study but may have led to the exclusion of relevant studies from other areas of health. Likewise, Dialnet tends to restrict results to publications in Spanish, which could limit the international representativeness of the review and may introduce linguistic selection bias. However, these limitations were minimized by the search in the other international databases PubMed and Scopus which are widely used for systematic reviews and include international studies in all areas of health.

In addition, considering the results obtained from different exercise modalities, greater benefits could be achieved through stronger consensus on outcome measurement and more precise management of exercise intensity, and encouraging discussion on the implementation of specialized rehabilitation and physical therapy teams within the context of chronic kidney disease. Moreover, it would be beneficial that future research further attention should be directed toward the effects of exercise on body composition to promote more comprehensive intervention approaches. Therefore, future lines of research should take all these aspects into account and use more homogeneous assessment tools, apply more homogeneous programmes and assess body mass as these are the evidence gaps that this study has found.

## 5. Conclusions

Based on the analysis of the studies included in this systematic review, it can be concluded that intradialytic exercise may reduce frailty levels in patients with chronic kidney disease through significant improvements in physical function and may also mitigate sarcopenia as evidenced by increases in muscle strength and enhanced performance in the sit-to-stand test. However, the results did not demonstrate significant changes in body composition.

The optimal exercise dosage appears to involve programs lasting 3 to 12 months, conducted two to three times per week, with aerobic exercise intensities ranging from 11 to 14 on the Borg scale and resistance training intensities between 10 and 13. Strength exercises should include 8 to 15 repetitions per set, with rest intervals of 40 to 60 s.

Despite the existing evidence, further studies are needed to explore this topic in greater depth, particularly to evaluate the impact of intradialytic exercise on quality of life among patients with chronic kidney disease.

**Clinical implications of the findings**

- Implementing intradialytic exercise programmes could be beneficial for patients with chronic kidney disease.
- Intradialytic exercise could improve frailty and sarcopenia in patients with chronic kidney disease.
- Intradialytic exercise programmes should be conducted two or three times per week, during 3 to 12 months and should include aerobic or resistance training to achieve better improvements.

## Figures and Tables

**Table 1:** Search Strategy for PubMed and Scopus.

Database	Search Strategy
**PubMed**	Haemodialysis *AND* physiotherapy. Haemodialysis *AND* physiotherapy were combined with muscle strength, exercise using the operator *AND*. Haemodialysis combined using the operator *AND* with exercise, weakness, muscle strength, functional physical performance, chronic kidney disease, hand grip strength. End-stage renal disease *AND* exercise and physiotherapy.
**Scopus**	Haemodialysis combined with the operator *AND* with the following words: short physical performance, hand grip strength, body mass index, exercise frailty phenotype fried gait speed test, physical performance, and exercise.
**PEDro**	Haemodialysis *AND* physiotherapy. Haemodialysis *AND* physiotherapy were combined with muscle strength, exercise using the operator *AND*. Haemodialysis combined using the operator *AND* with exercise, weakness, muscle strength, functional physical performance, chronic kidney disease, hand grip strength. End-stage renal disease *AND* exercise and physiotherapy.

**Table 2:** Studies collected.

Author	Mean Age	Sample Size	Type of Intervention	Assessment Tools	Results
Cardoso et al. [17]	CG = 49.4, GEA = 59.8, GEARF = 49.4	N = 59. CG = 20 (11M, 9H). GEA = 20 (9M, 11H). GEARF = 19 (10M, 9H).	CG: HD without exercise. GEA: Intradialytic aerobic cycling with cycle ergometer. Duration: 3 months. Sessions: 20 min, 3×/week. GEARF: Same as GEA but with blood flow restriction.	6 MWT. Static leg strength test.	Intradialytic cycling program with blood flow restriction produced a significant increase in distance walked in 6 min in the flow restriction group.
Krase et al. [18]	CG = 68.26, GEA = 66.24	N = 44. CG = 23 (13M, 10H). GEA = 21 (5M, 16H).	CG: Standard HD care. GEA: Intradialytic aerobic cycling with cycle ergometer. Duration: 4 weeks. Sessions: 60 min, 3×/week.	6 MWT. STS. Handgrip strength. Physical activity with pedometer. Muscle architecture with ultrasound.	Intradialytic cycling produced significant improvements in physical performance, 6 MWT, and handgrip strength in the exercise group.
Suhardjono et al. [19]	CG = 50.54, EG = 49.78, GEC = 46.38	N = 123. CG = 41 (21M, 18H). GEA = 42 (14M, 28H). GEC = 40 (18M, 21H).	CG: HD treatment. GEA: Aerobic cycling program. GEC: Intradialytic aerobic cycling and resistance exercise. No follow-up post-program.	Upper limb muscle strength with hand dynamometer. Lower limb muscle strength with hand dynamometer; 4 m gait speed. Skeletal muscle mass index via bioimpedance.	Aerobic intradialytic cycling generated significant improvements in lower limb strength in the exercise groups. No significant differences between exercise groups.
Yabe et al. [20]	CG = 75.3, GEC = 74.3	N = 83. CG = 19 (4M, 15H). GEC = 27 (13M, 14H).	CG: HD treatment. GEC: Aerobic exercise with cycle ergometer and resistance exercise with elastic bands. Duration: 1 year. Sessions: 30 min, 3×/week. No follow-up post-program.	Handgrip strength with dynamometer; 10 m gait speed. Lower limb strength with dynamometer. SPPB. GDS scale. Frailty: QMCOO questionnaire.	Control group experienced a significant decline in handgrip strength and gait speed. Exercise group showed no evidence of improvements in physical function.
Yabe et al. [21]	CG = 79, GEC = 78.7	N = 84. CG = 40 (19M, 21H). GEC = 44 (18M, 26H).	CG: HD with standard care. GEC: Aerobic cycling and resistance exercise with bands. Duration: 6 months. Sessions: 30–40 min, 3×/week. No follow-up post-program.	Lower limb strength with dynamometer. SPPB; 10 m gait speed.	Significant improvements in physical performance were obtained in the exercise group. No significant improvements in strength and gait speed between groups.
Zhang et al. [22]	CG = 62, GER = 60	N = 83. CG = 40 (16M, 27H). GER = 43 (18M, 26H).	CG: One HD session. GER: Progressive resistance exercises. Duration: 3 weeks. Sessions: 40–50 min, increased from 2 to 3×/week. No follow-up post-program.	6 MWT. STS. Handgrip strength with dynamometer.	Significant improvements were obtained in all variables measuring physical function.
Jamidshpour et al. [23]	CG = 58.46, GEC = 64.93	N = 28. CG = 13 (5M, 8H). GEC = 15 (3M, 12H).	CG: HD and habitual physical activity. GEC: Intradialytic aerobic cycling and moderate intradialytic resistance exercise. Duration: 2 months. Sessions: 1 h, 3×/week. No follow-up post-program.	6 MWT. Lower limb strength with dynamometer.	Exercise group obtained significant improvements in 6 MWT, but hip abductor and flexor strength did not improve. Control group decreased significantly.
Vogiatzaki et al. [24]	CG = 57.4, GEA = 58.1	N = 24. CG = 12 (4M, 8H). GEA = 12 (5M, 7H).	CG: HD and habitual physical activity. GEA: Intradialytic aerobic cycling. Duration: 6 months. Sessions: 1 h, 3×/week. No follow-up post-program.	BMI; 6 MWT.	Significant improvement in 6 MWT in the experimental group and compared to the control group. BMI showed no significant improvement.
Assawasaksakul et al. [25]	CG = 53.7, GEC = 52.5	N = 12. CG = 6 (3M, 3H). GEC = 6 (4M, 2H).	CG: HD with standard care. GEC: Intradialytic aerobic and resistance exercise with cycle ergometer. Duration: 6 months. Sessions: 1 h, 3×/week. No follow-up post-program.	6 MWT. Body composition: DXA. Daily activity measured with accelerometer. STS.	Physical activity increased in the experimental group compared to the control. Body mass did not increase in the exercise group but decreased in the control group.
Michou et al. [26]	CG = 54.5, GEC = 53.26	N = 29. CG = 14 (14H). GEC = 15 (15H).	CG: HD standard care. GEC: Combined aerobic and resistance exercise. Duration: 4 months. Sessions: 80–100 min, 3×/week.	6 MWT. STS. HGS. Body composition: BIA.	After the exercise program, significant improvements in strength, 6 MWT, and STS were evidenced.
Abdo et al. [27]	CG = 40, GEA = 40.5	N = 42. CG = 20 (11M, 9H). GEA = 22 (12M, 10H).	CG: HD treatment. GEA: Intradialytic aerobic exercise with cycle ergometer. Duration: 2 months. Sessions: 40 min, 3×/week.	Quadriceps muscle strength with dynamometer.	Quadriceps strength increased in the exercise group compared to the control group.
Yeh et al. [28]	CG = 53.91, GEC = 57.87	N = 62. CG = 32 (17M, 15H). GEC = 30 (11M, 19H).	CG: HD without exercise. GEC: Intradialytic exercise. Aerobic with cycle ergometer and resistance. Duration: 3 months. Sessions: 30 min, 3×/week.	6 MWT. STS-10. STS-60.	Increase in distance walked during 6 MWT in the exercise group compared to control. STS decreased time and increased number of repetitions in the exercise group compared to control.

Note: STS: sit-to-stand test; STS-10: time to perform 10 sit-to-stand repetitions; STS-60: number of sit-to-stand repetitions in 60 s; 6 MWT: 6 min walk test; HGS: handgrip strength measured with dynamometer; SPPB: short physical performance battery; DXA: dual-energy X-ray absorptiometry; CG: control group; EG: exercise group; GEA: aerobic exercise group; GEC: combined exercise group; QMCOO: Older Adult Health Questionnaire; M: male; H: female; HD: Hemodialysis.

**Table 3:** Methodological quality assessment of the articles according to the PEDro scale.

Authors	1	2	3	4	5	6	7	8	9	10	11	TOTAL
Cardoso et al. [17]	Y	Y	Y	Y	N	N	Y	Y	N	Y	Y	8 (good)
Krase et al. [18]	Y	Y	Y	Y	N	N	Y	Y	N	Y	Y	7 (good)
Suhardjono et al. [19]	Y	Y	Y	Y	N	N	N	Y	N	Y	Y	7 (good)
Yabe et al. [20]	Y	N	N	Y	N	N	N	N	N	Y	Y	4 (fair)
Yabe et al. [21]	Y	Y	Y	Y	N	N	N	N	N	Y	Y	6 (good)
Zhag et al. [22]	Y	Y	Y	N	N	N	Y	Y	Y	Y	Y	8 (good)
Assawasaksakul et al. [25]	Y	Y	Y	N	N	N	N	Y	Y	Y	Y	6 (good)
Jamshidpour et al. [23]	Y	Y	Y	Y	N	N	Y	Y	N	Y	Y	8 (good)
Vogiatzaki et al. [24]	Y	Y	Y	Y	N	N	Y	N	N	Y	Y	7 (good)
Michou et al. [26]	Y	Y	N	Y	N	N	N	N	N	Y	Y	5 (fair)
Abdo et al. [27]	Y	Y	N	Y	N	N	N	Y	N	Y	Y	6 (good)
Yeh et al. [28]	Y	Y	Y	Y	N	N	Y	N	N	Y	Y	7 (good)

The results of the methodological quality assessment using the PEDro scale indicate that 10 of the 12 articles included in this review have good methodological quality, with scores between 6 and 8. The remaining two articles [20,26] have a score of 4 and 5, respectively, indicating fair methodological quality. Y: Yes. The criterion is present. N: NO. The criterion is not present.

**Table 4:** Risk of bias assessment according to the Cochrane Risk of Bias tool.

	B1	B2	B3	B4	B5	B6	B7
Cardoso et al. [17]	+	+	+	+	-	+	+
Krase et al. [18]	+	+	+	+	-	+	+
Suhardjono et al. [19]	+	+	+	U	U	+	+
Yabe et al. [20]	-	-	+	-	+	-	+
Yabe et al. [21]	+	-	-	-	-	+	+
Zhang et al. [22]	+	+	+	+	+	+	+
Assawasaksakul et al. [25]	+	+	+	U	+	+	+
Jamshidpour et al. [23]	+	+	+	+	+	+	+
Vogiatzaki et al. [24]	+	+	+	+	-	+	+
Michou et al. [26]	+	U	U	U	+	+	+
Abdo et al. [27]	+	U	U	+	+	+	+
Yeh et al. [28]	+	+	+	U	+	+	+

**Note:** B1 = Selection bias due to inadequate sequence generation; B2 = Selection bias due to inadequate allocation concealment; B3 = Performance bias due to lack of blinding of participants; B4 = Detection bias due to lack of blinding of outcome assessors; B5 = Attrition bias due to incomplete outcome data; B6 = Reporting bias due to incomplete selective reporting; B7 = Other sources of bias; U = Unclear. +: Low risk of bias; -: High risk of bias.

## Data Availability

Data is available upon reasonable request to the authors.
